# Catheter Ablation of Long-Lasting Accelerated Idioventricular Rhythm in a Patient with Mild Left Ventricular Dysfunction

**DOI:** 10.1155/2012/143864

**Published:** 2012-07-26

**Authors:** Takanao Mine, Mamoru Hamaoka, Hideyuki Kishima, Tohru Masuyama

**Affiliations:** Cardiovascular Division, Department of Internal Medicine, Hyogo College of Medicine, 1-1 Mukogawa-cho, Nishinomiya 663-8501, Japan

## Abstract

A 35-year-old woman with long-lasting accelerated idioventricular rhythm (AIVR) exhibited palpitation and dyspnea on exertion and mild left ventricular (LV) dysfunction during followup. Symptoms appeared 10 years after the AIVR was first noted, and she underwent catheter ablation for curative therapy of AIVR after 12 years. Radiofrequency ablation of the anteroseptal site of the LV at the earliest activation terminated rhythm. An echocardiogram, taken 1 month after discharge, subsequently revealed that the left ventricular wall motion had normalized. This is a rare case of long-lasting AIVR with mild LV dysfunction.

## 1. Introduction

Accelerated idioventricular rhythm (AIVR) is a ventricular rhythm consisting of three or more consecutive monomorphic beats with 50–100 beats per minute. AIVR emerges and terminates gradually. It generally manifests in acute myocardial infarction, digitalis toxicity, and valvular disease [1–3]. In particular, in patients with acute myocardial infarction, AIVR is often observed during reperfusion or within two days after the onset. It is rarely observed in patients with normal hearts. We experienced a case with AIVR that lasted for more than 12 years without heart disease. 

## 2. Case Report

**Figure 1:**
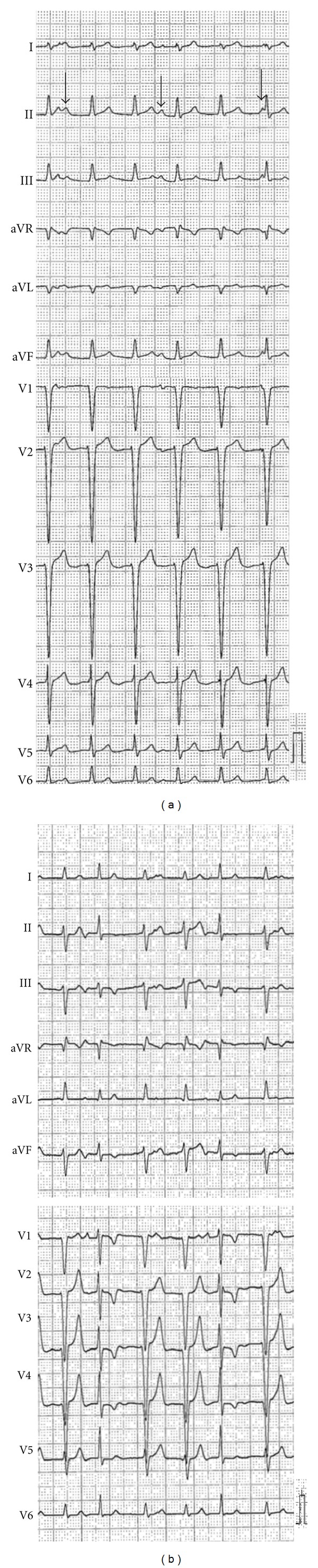
(a) The baseline 12-lead electrocardiogram (ECG) in the supine position shows accelerated idioventricular rhythm (AIVR) without atrioventricular conduction. The arrows indicate the P wave. (b) The ECG in the standing position immediately after exercise. The waveforms of limb leads in the standing position differ from those in the supine position.

This 35-year-old woman had been informed of electrocardiogram (ECG) abnormalities that showed continuous idioventricular rhythm (heart rate 75 bpm) after a physical examination at age 23. Subsequently, similar ECG findings were noted during routine annual medical examinations. From the age of 32, she became aware of palpitation and dyspnea on exertion. Beta-blockers were ineffective. At age 35, the ECG showed AIVR, with a heart rate of 103 bpm and a QRS width of 100ms (Figure 1(a)), and Holter ECG revealed that the AIVR persisted throughout the day (heart rate 87–147/min, mean heart rate 108/min). Transthoracic echocardiography showed mild left ventricular dysfunction, left ventricular ejection fraction (LVEF) of 51% and left ventricular end-systolic diameter (LVDd) of 51 mm. During the treadmill exercise test (TET), the AIVR rate gradually increased to 160/min, and the ECG after the exercise showed only a few beats with atrioventricular conduction beats and a QRS width of 80 ms (Figure 1(b)). The TET was terminated at nine minutes due to dyspnea. She was referred to undergo catheter ablation for drug-refractory AIVR. 

**Figure 2:**
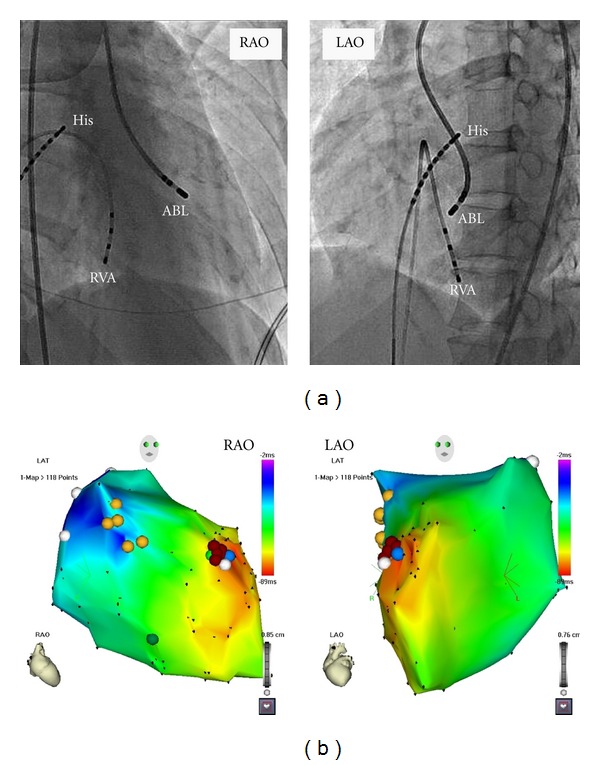
Fluoroscopic images (a) and 3D electroanatomic map (b) of the LV demonstrating the successful ablation site. ABL: ablation catheter; His; His bundle catheter; RVA: right ventricular apex; RAO: right anterior view; LAO: left anterior view.

**Figure 3:**
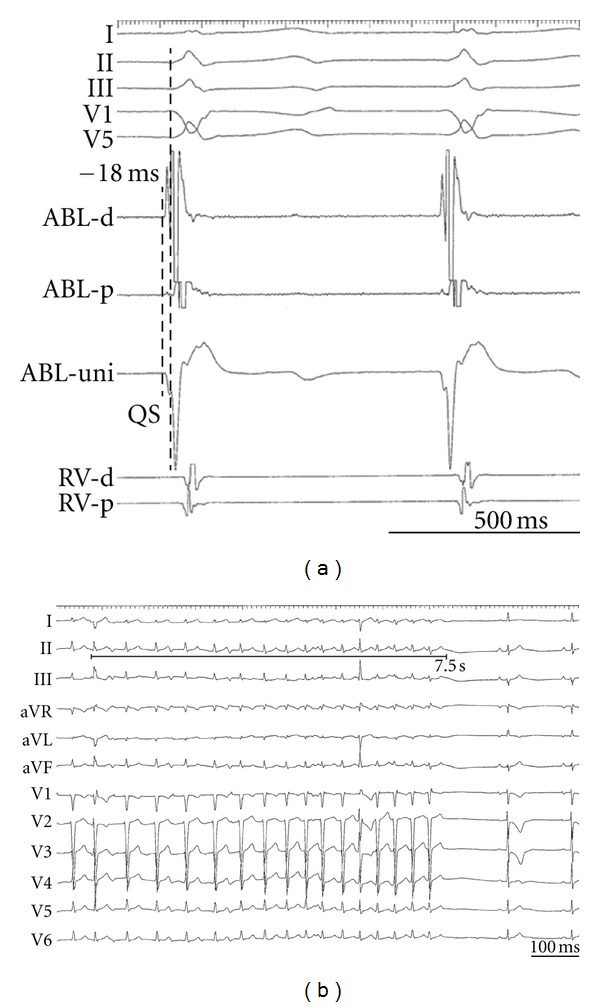
(a) The local contact bipolar and unipolar electrograms at the ablation site during AIVR. In the bipolar recording, the potential preceded the surface QRS wave by 18 ms. The unipolar recording exhibited a QS pattern. (b) The intracardiac electrogram during the RF delivery. The RF current interrupted the AIVR.

After obtaining informed consent from the patient, the electrophysiological study and catheter ablation were performed. Multipolar electrode catheters were positioned at the high right atrium (HRA), His bundle recording area, and the right ventricular apex (RVA), via the right femoral vein. One 4 mm tip deflectable 7 Fr catheter (Navister) was inserted via the right femoral artery for mapping and ablation. We found constant atrioventricular conduction during HRA pacing (cycle length 650 ms). The AIVR was not terminated by rapid RVA pacing. The activation map of the left ventricle (LV) during AIVR using an electroanatomical mapping system (CARTO, Biosense-Webster) revealed the focus of the AIVR (Figure 2(b)) at the anteroseptal site of the LV (Figure 2(a)). RF energy application (power of 30 W with a target temperature of 55°C) at the site of the earliest activation was successful in terminating the rhythm (Figure 3). The patient was restored to sinus rhythm immediately, and tachycardia was not inducible at the end of the study. An echocardiogram, taken 1 month after discharge, revealed that the left ventricular wall motion was normalized, LVEF of 63% and LVDd of 47 mm, and TET was performed for 14 minutes without symptoms. The patient had no recurrence of tachycardia during followup up to 6 months.

## 3. Discussion

We have encountered cases of mild heart dysfunction and symptoms in patients with long-lasting AIVR. The QRS waveforms of AIVR show a broader QRS width than sinus rhythm. Ventricular automaticity usually occurs at 30–40 beats/min. AIVR is clearly faster than ventricular automaticity and obviously slower than ventricular tachycardia. The definition of heart rate has been reported differently [1]. In this case, the heart rate was 87–147 bpm and which was obviously faster than ventricular automaticity, and the QRS width was broader than the sinus rhythm by 100 ms. The ECG had shown abnormal findings for the past 12 years. Thus, this case was diagnosed as AIVR lasting for more than 12 years. The mechanism of AIVR is unknown. The rhythms appear frequently in the early stage of acute myocardial infarction. Therefore, they might be the result of enhanced automaticity in abnormal Purkinje fibers [4]. The rhythm begins gradually with long-coupling ventricular beats. The main electrophysiological mechanism is considered to be due to abnormal calcium-dependent automatism in the Purkinje fibers [4]. The site of origin appears to be in the region of the anterior fascicle and there is a potential on the ablation catheter at the successful site. Some cases are ameliorated by treatment with a beta blocker [4]. 

AIVR usually manifests in acute myocardial infarction, digitalis toxicity, and valvular disease [1–3]. It is rarely seen in patients with normal hearts. AIVR is an essentially benign and self-limited condition [2, 3, 5]. There have been some cases that required medication [5] and none that required catheter ablation, particularly in young infants. There have been reports of cases in which catheter ablation is necessary. This is a rare case of drug-refractory AIVR that required catheter ablation.

The cause of cardiac dysfunction in this case was not clear. In most cases, AIVR is an ectopic ventricular rhythm with ≥3 consecutive ventricular premature beats with a rate faster than the normal sinus rhythm, but slower than ventricular tachycardia (less than 100/min). It is uncommon that AIVR lasts for a long time, as in this case. One cause of cardiac dysfunction might be the long-lasting rhythm. Tachycardia-induced cardiomyopathy (TICMP) can be considered as a cause of impaired cardiac function. TICMP is characterized by ventricular dysfunction resulting from an increased ventricular rate [6, 7]. TICMP can usually be seen in tachycardia due to atrial fibrillation or atrial flutter. The definition of TICMP is not clearly determined. It is often defined when the LVEF drops below 50% and cardiac function improves after 6 months of treatment for the tachycardia [6, 7]. Considering the improvement of LVEF 1 month after the treatment of AIVR, the mechanism of the mild left ventricular dysfunction may be due to TICMP.

AIVR patients generally have a good prognosis. It is rare to discover left ventricular dysfunction in a patient with AIVR during followup. However, in such cases the long-lasting symptoms might appear or AIVR may cause cardiac dysfunction. The long-lasting AIVR in this case should be considered for catheter ablation therapy. In any case, further study is necessary in consideration of cases with AIVR.
